# Bipolar versus monopolar transurethral resection of the prostate for benign prostatic hyperplasia: safe in patients with high surgical risk

**DOI:** 10.1038/srep21494

**Published:** 2016-02-19

**Authors:** Er J. Yang, Hao Li, Xin B. Sun, Li Huang, Li Wang, Xiao X. Gong, Yong Yang

**Affiliations:** 1 Department of Urology,Taihe Hospital, Hubei University of Medicine, Hubei, China

## Abstract

Here, we compared the effects of bipolar and monopolar transurethral resection of the prostate (B-TURP, M-TURP) for treating elderly patients (≥75 years) with benign prostatic hyperplasia(BPH) who had internal comorbidities. Eligible BPH patients were aged ≥75 years and had at least one internal comorbidity. In this open-label, prospective trial, patients were assigned to B-TURP (n = 75) and M-TURP (n = 88) groups. Data on prostate volume (PV), urination, and time during perioperative period were compared; data associated with urination and complications at one year postoperatively were also compared. Finally, follow-up data were available for 68 and 81 patients in the B-TURP and M-TURP group, respectively. No deaths were recorded. Intraoperative bleeding was lower and irrigation time, indwelling catheter time, and hospital stay were shorter in the B-TURP group than in the M-TURP group (p < 0.001). No difference was observed with respect to operation time (p = 0.058). At one year after the operation, differences with respect to urination and complications were not significant. In conclusion, Short-term efficacy of B-TURP or M-TURP was satisfactory for elderly patients with BPH who had internal comorbidities. Besides, B-TURP is a more sensible choice because it has a lower prevalence of adverse effects.

Benign prostatic hyperplasia (BPH) is a common disease in elder men. It may cause urinary urgency, frequency, nocturia, dysuria, and complications such as urinary tract infection, bladder stones, and hydronephrosis that sharply affect the quality of life (QOL). Therefore, prompt and effective interventions are necessary for treating BPH. In some patients with BPH, especially high-risk patients with severe symptoms, conventional drug treatment does not significantly improve lower urinary tract symptoms (LUTS). Usually, surgery is needed to achieve satisfactory outcomes. In recent times, traditional open resection of the prostate is gradually being replaced by minimally invasive, safe, and effective methods such as monopolar transurethral resection of the prostate (M-TURP), bipolar transurethral resection of the prostate (B-TURP), and greenlight photoselective vaporization of the prostate (PVP). These endoscopic techniques provide large benefits to patients with BPH, particularly elderly patients who are unable to tolerate an open surgery. Owing to the high-risk, previous studies comparing the effects of these minimally invasive techniques have included patients without serious comorbidities^1^,^2^,^3^. Therefore, many elderly patients with serious comorbidities have no choice but to live with a poor QOL.

In the current study, we prospectively compared the clinical efficacy and safety of B-TURP and M-TURP for treating elderly patients with high surgical risk.

## Material and Methods

### Patients

The study continually recruited patients with BPH who were diagnosed at the Department of Urology, Taihe Hospital, Hubei University of Medicine (Taihe, China) between Feb 1, 2012, and Apr 31, 2015. Elderly patients with high surgical risk were defined as patients aged ≥75 years that experienced at least one internal comorbidity, e.g., hypertension or diabetes. Patients with BPH were included in the study if 1) they were aged ≥75 years and had high surgical risk; 2) they regularly used α-receptor blockers, 5α-reductase inhibitors, and/or M-receptor blockers for more than six months but did not achieve a satisfactory international prostate symptom score (IPSS); and 3) they were willing to undergo B-TURP or M-TURP and provided written informed consent. Patients with 1) documented or suspected prostate cancer (PCa), bladder stone or diverticula, neurogenic bladder, or urethral stricture; 2) any one internal comorbidity that deteriorated significantly in the past three months, or 3) clinical suspicion of tumors in other body parts were excluded from the study. All persons gave their informed consent prior to their inclusion in the study. The trial was approved by the Ethics Committee of Taihe Hospital and he methods were carried out *in accordance with* the approved guidelines. This was not a registered trial.

### Classification

Patients were assigned centrally at the Department of Urology, Taihe Hospital, Hubei University of Medicine (Taihe, China), and each eligible patient was interviewed. The details of the two surgical methods, such as benefits and drawbacks, risk, and expected expenses, were fully explained to the eligible patients. Next, the patients were given suggestions based on their specific condition. However, the surgical method, i.e., B-TURP or M-TURP, was eventually decided by the patients. The type of surgical method selected was known to both the patient and his/her surgeon.

### Preoperative preparation

In addition to routine examinations, following measures were taken for patients with different internal comorbidities before they underwent either B-TURP or M-TURP: 1) controlling blood pressure within 140/90 mmHg in hypertensive patients; 2) maintaining fasting blood glucose at 6–8 mmol and 2-h postprandial blood glucose below 11 mmol/L; 3) improving and maintaining pulmonary or cardiac function in patients with chronic bronchitis, emphysema, or cardiac dysfunction; and 4) controlling blood pressure or treating anemia with active symptomatic treatment in patients with chronic renal insufficiency. Patients with deteriorating or unstable comorbidities were transferred to other departments for further treatment before being reconsidered for inclusion in the study.

### Surgical procedure

A TURis system (Olympus, Tokyo, Japan) was used for treating patients in the B-TURP group (280 W for cutting and 100 W for coagulation; 0.9% NaCl as irrigation fluid) and an M-TURP system (Storz, Tübingen, Germany) was used for treating patients in the M-TURP group (130 W for cutting and 50 W for coagulation; Glycine as irrigation fluid). All the patients were placed in the lithotomy position and were given general anesthesia with propofol. The two surgical procedures were performed based on the methodology described by Fayad *et al.*^4^ under intravenous anesthesia with propofol. Bladder irrigation was initiated immediately after the patient was transferred to a ward or intensive care unit (ICU).

### Outcome measures and follow-up

The following data were collected before the surgery and at one year after the surgery: a) IPSS, b) maximum flow rate (Q_max_), c) post-void residual (PVR), and d) QoL. Preoperative prostate volume (PV) was measured by performing B-ultrasonic examination. The following data were obtained after the surgery: a) operation time, b) perioperative bleeding, c) bladder irrigation time, d) indwelling catheter time, and e) duration of hospital stay. Data related to surgical complications like a) secondary bleeding, b) urethrostenosis, c) enuresis, and d) BPH relapse were obtained at one year after the surgery. All the patients were asked to visit our department at any time if they experienced any discomfort.

### Statistical analysis

SPSS software version 13.0 was used for data analysis. Discrete and continuous variables were compared using chi-square test and *t* test, respectively. P values less than 0.05 were considered statistically significant.

## Results

In all, 163 patients were included in the study. These patients were aged 75–90 years (mean age, 81.66 ± 4.03 years). All the patients had IPSS ≥15 and QoL of ≥4^5^. The main comorbidities were hypertension in 128 patients, diabetes in 85 patients, chronic bronchitis/emphysema in 80 patients, cardiac dysfunction in 42 patients, chronic renal insufficiency in 25 patients, and pacemaker in 9 patients.

Before the surgical procedure, nine patients with systolic blood pressure (BP) at 160–180 mmHg and two patients with cardiac dysfunction were transfer to Department of Cardiology because their situation was difficult to control. After specialized care, their systolic BP level was all controlled under 150 mmHg for the nine patients; the edema of lower extremity and discomfort of the precordial area for the two patients was obviously relieved. One patient with both hypertension (150~160/80~90 mmHg) and chronic renal insufficiency (creatinine 200~220 umol/L, urine protein 0.8~1.0 g/d) was transferred to Department of Nephrology. After six weeks’ treatment, BP (130~140/70~80 mmHg) and urine protein (0.1~0.2 g/d) both were relieved to a satisfactory level.

After the procedure, corresponding symptomatic treatment was taken for patients with different comorbidities, like what we did preoperatively. One patient with chronic bronchitis occurred dyspnea immediately at the 1^st^ night and was transferred to ICU immediately. After two day’s enhance care, his symptom had an obvious improvement. For the patient transferred to Department of Nephrology preoperatively, the creatinine level had a transient rise at the 3^rd^ day (peak value 420 umol/L) and decreased gradually to the level around 200 umol/L. No deaths or other emergency occurred in this patient population.

After a follow-up of one year, complete data were available for 149 patients (68 in the B-TURP arm and 81 in the M-TURP arm), which gave a follow-up rate of 91.4%. Of the remaining 14 patients who were lost to follow-up, eight refused to come back to our department for further consultation until the closure of the study and six could not be contacted or provided incomplete data over telephone calls.

Table 1 shows the baseline characteristics of the 149 patients for whom complete data were available. No significant differences were observed in the baseline data of patients in the two arms, except for QOL score. Data obtained during the perioperative period are summarized in Table 2. Bleeding volume was significantly lower in the B-TURP arm than in the M-TURP arm. Irrigation time, indwelling catheter time, and duration of hospital stay were significantly higher in the M-TURP arm than in the B-TURP arm. Data on urination and complications obtained one year after the surgery are summarized in Tables 3 and 4, respectively. No significant differences were observed between two arms in all the data.

## Discussion

Epidemiological data show that approximately 70% men aged >70 years experience different degrees of BPH symptoms^6^. Particularly, patients with BPH having various chronic internal complications have high surgical risk. Although M-TURP has been considered as the gold standard for treating BPH over the past decade, it is associated with complications. A study^7^ showed that, after one month of the M-TURP procedure, incidence rates of complications, transurethral resection syndrome (TURS), and mortality were 18%, 2%, and 0.2%, respectively. On the other hand, the M-TURP system used in our study has a bipolar circuit and uses low temperature at the tissue surface (40 °C–70 °C) that allows less exfoliation of the wounded surface and less hemorrhage. In addition, because of the less evaporation of the prostate tissue, it is possible for surgeons to collect specimens for further pathological examination, which improves the detection of incidental PCa. Moreover, the using of saline as the irrigation solution during the operation, which has lower influence on plasma osmotic pressure compared to M-TURP (Glycine as irrigation fluid), thus significantly reducing the incidence of TURS.

To the best of our knowledge, no prospective studies have compared the clinical efficacy and safety of B-TURP and M-TURP in elderly patients (≥75 years) with BPH who had internal comorbidities to date. The oldest patient cohorts in the published data came from the study of Michielsen *et al.*^8^, with an age of 73.8 and 73.1 for the B-TURP and M-TURP arms, respectively. A latest systematic review and meta-analysis of the clinical effectiveness of B-TURP compared with M-TURP conducted by Omar *et al.*^9^ found that no statistically significant differences in terms of IPSS or QoL score. Besides, although the Qmax were significant at 3, 6 and 12 months, but no clinically significant differences were found. Meanwhile, the adverse events, e.g., TURS, blood transfusion, and clot retention, were significantly fewer for B-TURP. It provided the evidence-based evidence that B-TURP had the same effect with M-TURP, also accompanying a higher safety. Our study also showed that both B-TURP and M-TURP can significantly improve the LUTS of patients with BPH effectively for elderly patients with high surgical risk. IPSS and other measures associated with urination at one year after the surgery were markedly improved compared with the values at baseline, and no significant differences were observed between the two arms. The rate of complications at one year after the surgery was also not significantly different between the two arms.

The operation time of M-TURP was slightly shorter than that of B-TURP, but without statistic or clinical differences. The prevalence of TURS was significantly higher for M-TURP and no TURS occurred in B-TURP arm. Except this, all other data (amount of bleeding, bladder irrigation time, indwelling catheter time, and duration of postoperative hospital stay) significantly favored B-TURP over M-TURP (p < 0.05). These advantages were observed mostly due to a better hemostatic effect of the B-TURP. A shorter duration of hospital stay and a faster post-operative recovery could also reduce the financial burden, particularly in the developing country like China. Based on the advantages mentioned above, for patients with a poor general condition or with a serious comorbidity, it is wise to choose B-TURP, which reduces the adverse effects of double trauma from both the comorbidity and surgery.

### Limitations

However, long-term durability of both B-TURP and M-TURP for elderly patients with high surgical risk needs to be investigated further because the follow-up duration of this study was only one year. A four-year follow-up study conducted by Autorino *et al.*^10^ showed that the significant improvements in both groups were maintained at 4 yr for the IPSS, QoL score, Qmax, and PVR versus baseline values. Only 2 of 32 (6.2%) in B-TURP group and 3 of 31 (9.6%) in M-TURP patients required reoperation because of late complications (p = 0.15). The other limitation of our study is the study design. The non-randomized, open-labeled design of this study may lead to potential bias. As we can see, the age and QoL score at baseline were not comparable for patients with successful follow up between two groups. Therefore, further large-scale, rigorous, randomized controlled trials should be performed to determine the differences between B-TURP and M-TURP for treating patients with BPH who have high surgical risk.

## Conclusions

In conclusions, either B-TURP or M-TURP can be used effectively for treating elderly patients with BPH who have a high surgical risk by stabilizing comorbidities, carefully performing the operation, and carefully monitoring patients’ condition. Besides, B-TURP is a more sensible choice for patients with a poor general condition or with serious comorbidities because it has a lower prevalence of adverse effects.

## Additional Information

**How to cite this article**: Yang E. J. *et al.* Bipolar versus monopolar transurethral resection of the prostate for benign prostatic hyperplasia: safe in patients with high surgical risk. *Sci. Rep.* **6**, 21494; doi: 10.1038/srep21494 (2016).

## Figures and Tables

**Table 1:** Baseline data of 149 patients with successful follow up.

	B-TURP (68 cases)	M-TURP (81 cases)	P
Age, yr	82.71 ± 4.22	80.68 ± 3.64	0.025
PV (ml)	53.00 ± 6.81	51.30 ± 6.96	0.275
IPSS	25.63 ± 2.79	26.91 ± 3.76	0.088
QoL	4.89 ± 0.80	5.24 ± 0.70	0.042
Qmax (mL/s)	6.72 ± 1.10	7.29 ± 1.77	0.093
PVR (ml)	126.63 ± 17.84	120.93 ± 15.58	0.133

Data are showed as means ± standard deviations. All p-values are nonsignificant except for age and QoL score.

**Table 2:** Comparisons of data during the perioperative period.

groups(cases)	operation time (min)	bleeding (ml)	irrigation time (h)	Indwelling catheter time (d)	hospital stay (d)	TURS*
B-TURP (n = 75)	65.00 ± 6.99	79.18 ± 9.70	24.29 ± 1.62	4.00 ± 0.37	7.27 ± 0.65	0
M-TURP (n = 88)	62.40 ± 6.10	115.29 ± 8.61	42.71 ± 1.99	5.19 ± 0.61	8.38 ± 0.70	6
P	0.058	<0.001	<0.001	<0.001	<0.001	0.027

^*^2 cells (50.0%) have excepted count less than 5, so Fish’s Exact Test was used rather than Pearson Chi-Square Test.

**Table 3:** Follow up data associated with urination at one year postoperatively.

	B-TURP (n = 68)	M-TURP (n = 81)	P*
IPSS	5.53 ± 1.08	5.83 ± 0.80	0.160
QoL	1.92 ± 0.82	2.17 ± 0.67	0.140
Qmax (ml/s)	18.68 ± 2.47	17.89 ± 1.58	0.091
PVR (ml)	24.44 ± 3.75	22.80 ± 4.11	0.069

^*^All p-values are nonsignificant.

**Table 4:** Data associated with complications at one year postoperatively*.

groups(cases)	Secondary bleeding	urethrostenosis	enuresis	BPH relapse
B-TURP (n = 68)	1	2	2	2
M-TURP (n = 81)	2	3	2	1
P	1.000	1.000	1.000	0.606

^*^Fish’s Exact Test was used for all the measures in this table.
